# Characterization of the retinal vasculature in fundus photos using the PanOptic iExaminer system

**DOI:** 10.1186/s40662-020-00211-5

**Published:** 2020-09-08

**Authors:** Huiling Hu, Haicheng Wei, Mingxia Xiao, Liqiong Jiang, Huijuan Wang, Hong Jiang, Tatjana Rundek, Jianhua Wang

**Affiliations:** 1 Shenzhen Key Laboratory of Ophthalmology, Shenzhen Eye Hospital, Jinan University, Shenzhen, China; 2 Department of Ophthalmology, Bascom Palmer Eye Institute, University of Miami Miller School of Medicine, 1638 NW 10th Avenue, McKnight Building - Room 202A, Miami, FL 33136 USA; 3 Department of Electrical and Computer Engineering, Florida International University, Miami, FL USA; 4 School of Electrical and Information Engineering, North Minzu University, Yinchuan, Ningxia China; 5 Eye hospital, China Academy of Chinese Medical Sciences, Beijing, China; 6 Department of Neurology, University of Miami Miller School of Medicine, Miami, FL USA

**Keywords:** Smartphone ophthalmoscope, Image analysis, Deep learning, Arteriovenous ratio, Vessel density, Retina

## Abstract

**Background:**

The goal was to characterize retinal vasculature by quantitative analysis of arteriole-to-venule (A/V) ratio and vessel density in fundus photos taken with the PanOptic iExaminer System.

**Methods:**

The PanOptic ophthalmoscope equipped with a smartphone was used to acquire fundus photos centered on the optic nerve head. Two fundus photos of a total of 19 eyes from 10 subjects were imaged. Retinal vessels were analyzed to obtain the A/V ratio. In addition, the vessel tree was extracted using deep learning U-NET, and vessel density was processed by the percentage of pixels within vessels over the entire image.

**Results:**

All images were successfully processed for the A/V ratio and vessel density. There was no significant difference of averaged A/V ratio between the first (0.77 ± 0.09) and second (0.77 ± 0.10) measurements (*P* = 0.53). There was no significant difference of averaged vessel density (%) between the first (6.11 ± 1.39) and second (6.12 ± 1.40) measurements (*P* = 0.85).

**Conclusions:**

Quantitative analysis of the retinal vasculature was feasible in fundus photos taken using the PanOptic ophthalmoscope. The device appears to provide sufficient image quality for analyzing A/V ratio and vessel density with the benefit of portability, easy data transferring, and low cost of the device, which could be used for pre-clinical screening of systemic, cerebral and ocular diseases.

## Background

The retina provides a direct, non-invasive, and easily accessible window for observing the microvascular system. The vasculature of the retina and brain are anatomically and physiologically similar [1]. Fundus photography is a useful tool to observe and monitor changes in the retinal vasculature. Large-scale epidemiology studies based on fundus photography reported that the changes of retinal vessels are associated with the risk of retinal and systemic diseases, including diabetic retinopathy [2], stroke [3], cardiovascular mortality [4] and dementia [5].

While the traditional fundus camera offers good-quality images for analysis of large retinal vessels, these fundus camera systems are based in hospitals and research facilities. The office-based systems require skillful technicians and are limited to the subjectivity of the clinician’s interpretation. These systems are also bulky and costly, which limits their use in community screening efforts, especially in remote areas [6]. Two recent remarkable breakthroughs are improvements in teleophthalmology and smartphone adapted devices. With the advantage of easy image capturing, easy data transferring, and low cost, the PanOptic ophthalmoscope adapted with a smartphone and data acquisition application (iExaminer) may provide an ideal solution for pre-clinical disease screening [7–9]. The PanOptic ophthalmoscope system has been used in some previous studies. However, none of these previous studies quantified retinal vasculature, which renders whether the system can be used to characterize retinal vasculature. The goal of the present study was to characterize retinal vasculature by quantitative analysis of arteriole-to-venule (A/V) ratio and vessel density in smartphone-acquired fundus photographs using the PanOptic iExaminer System.

## Methods

### Subject selection

This study was approved by the Institutional Review Board (IRB) of the University of Miami (ID: 20070492), and all study subjects were treated according to the tenets of the Declaration of Helsinki. A signed written informed consent was obtained from each subject. Ten subjects were recruited at the Bascom Palmer Eye Institute. A total of 19 eyes were imaged. One study subject agreed to have one eye dilated for the present study. The following exclusion criteria were used: shallow anterior chamber, ocular surgery history, refractive errors more than ±6.0 diopters, or any systemic inflammatory or infectious diseases.

### Image capture

**Fig. 1:**
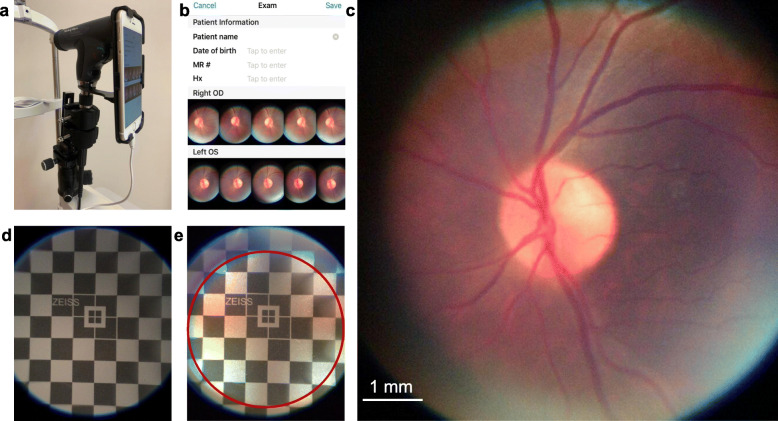
PanOptic iExaminer System and field of view calibration. **a** PanOptic ophthalmoscope adapted with a smartphone (iPhone 6 s) was mounted on a slit lamp base with the chinrest. **b** iExaminer software was used for image acquisition. **c** A representative fundus photo was captured using the system. **d** The field of view (without internal illumination) was tested using the Zeiss field of view calibration tool placed in front of the PanOptic ophthalmoscope under room light. **e** The field of view was calibrated as 7.0 mm × 7.0 mm with the emitted light on

PanOptic ophthalmoscope (WelchAllyn, Skaneateles Falls, NY) was mounted on a slit-lamp base to facilitate the alignment of the system with the eye (Fig. 1). An external fixation target, placed in front of the contralateral eye, was also provided. The PanOptic ophthalmoscope was adapted to the iExaminer adapter (WelchAllyn, Skaneateles Falls, NY). Fundus images were captured by the iExaminer Pro application with the iPhone 6 s. A short video clip was recorded during acquisition; then, the five best images were manually selected based on the focus of vasculature and optimal visualization (Fig. 1). For the field of view calibration, a Zeiss field of view calibration tool (S/N4007) was placed in front of the ophthalmoscope under room light (Fig. 1). The field of view was calibrated to 7.0 mm × 7.0 mm with the emitted light on. Each eye was dilated with topical tropicamide 1.0%. During imaging in a dark test room, the subject was seated, and the chin rested on the chinrest while looking at the fixation target.

### A/V ratio analysis

**Fig. 2:**
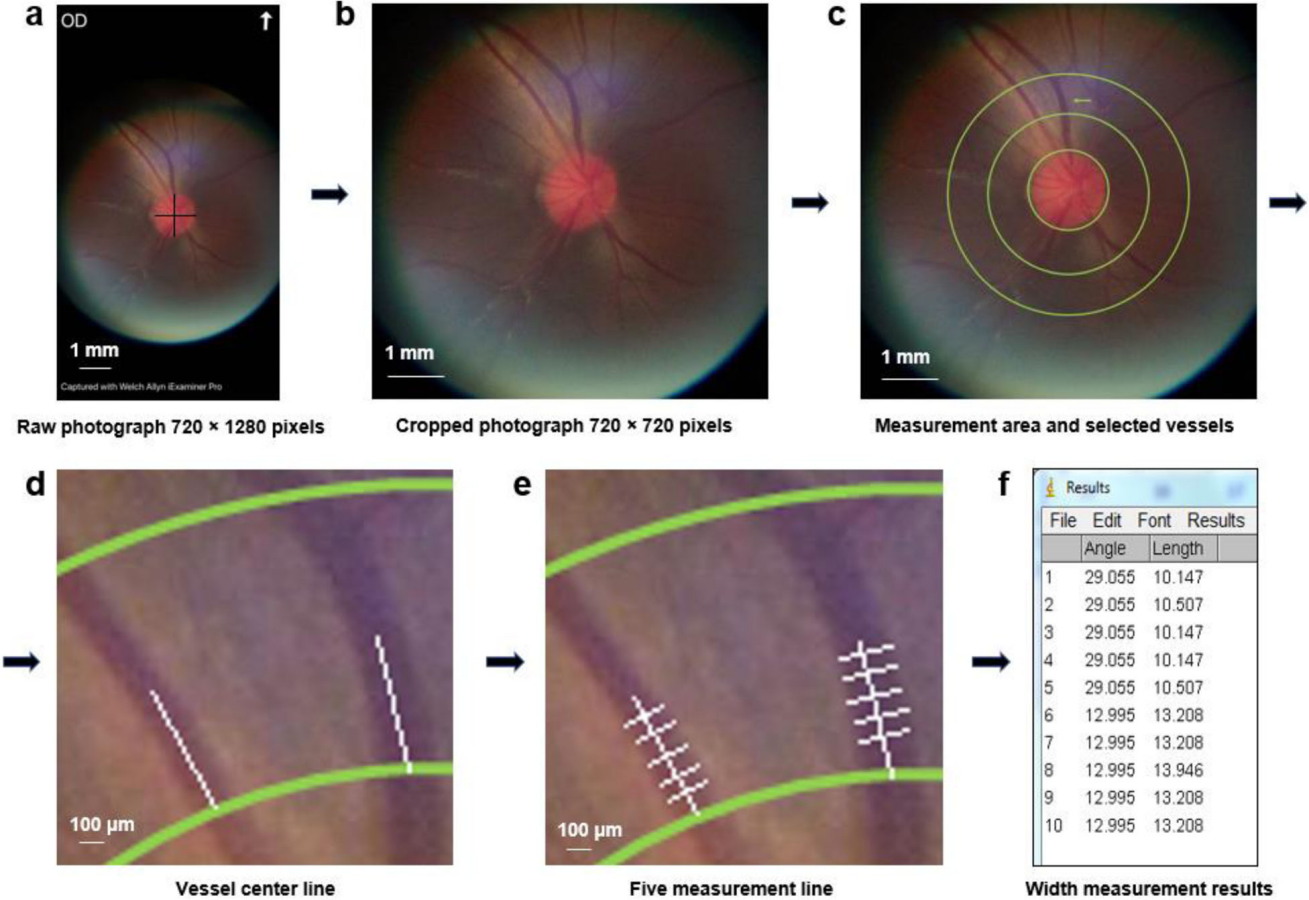
Example of arteriole and venule diameter measurements. **a** Raw fundus photograph acquired using the Panoptic iExaminer system. **b** Cropped square photograph. **c** The measurement area was within 0.5 to 1 disc diameter of the edge of the disc margin. The arrow showed the selected parallel arteriole and venule for demonstration. **d** Vessel centerline was drawn along with the long vessel axis. **e** Five non-overlapping measurement lines for each vessel were made. **f** The width of each measurement line was calculated using ImageJ

The exported raw image was 1024 (vertical) × 720 (horizontal) pixels. To make the image conform to a square ratio, the black portions on the top and bottom of the image were removed, and the raw image was trimmed to 720 × 720 pixels with a field of view of 7.0 × 7.0 mm (Fig. 2). The measurement area for arteriole and venule was done within 0.5 to 1 disc diameter from the edge of the disc margin, as done in previous studies [10, 11]. The selected parallel arteriole and venule diameters were measured using the straight-line measurement tool of ImageJ. Five non-overlapping measurements for each vessel were made (Fig. 2). The diameters were then averaged, and the arteriole to venule ratio was calculated. Two photographs with the best focusing and centration of the optic nerve head of each eye were selected and calculated. The measurement for the A/V ratio was performed by two researchers.

### Vessel extraction using deep learning U-NET and density analysis of retinal vessels

**Fig. 3:**
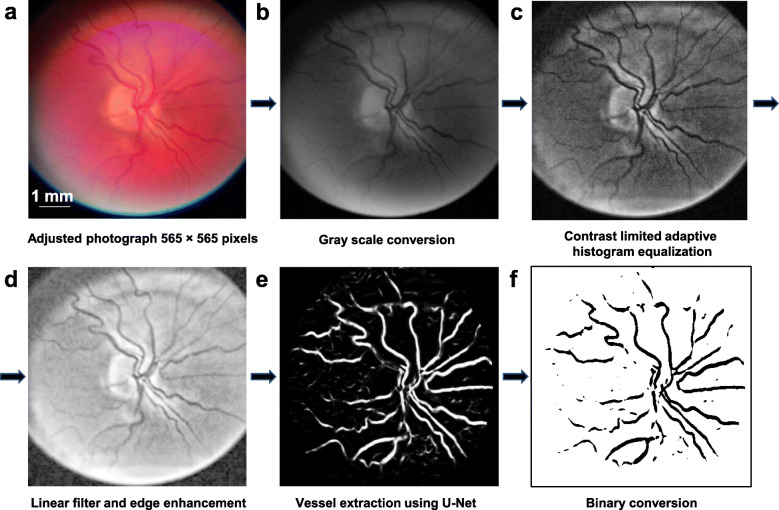
Example of retinal vessel density analysis. **a** Cropped square photograph. **b** Grayscale conversion using Gray = MAX Contrast. **c** Contrast enhancement using contrast limited adaptive histogram equalization. **d** Vessel border enhancement using the linear filter and edge enhancement. **e** Vessel extraction using U-Net. **f** Vessel binary conversion using ImageJ

We developed the image processing codes in the MATLAB (MathWorks, Natick, MA) for image conversion and filtering. We also developed U-Net image segmentation in Python (ver. 3.6.5). The photograph was converted into a square with a resolution of 565 × 565pixels (Fig. 3) and then transformed into the grayscale format using Gray = MAX Contrast (R, G, B). Although the green channel is commonly used to convert RGB images to grayscale, we used all channels to convert the RGB images so that they would contain more of the red color in these fundus photos to preserve more details of the vessels. The weight of each channel was obtained from the training set (DRIVE), and the setting was red × 0.299 + green × 0.587 + blue × 0.114. The image was then processed by contrast enhancement using contrast limited adaptive histogram equalization (CLAHE). We used the function of *adapthisteq* with NumTiles (25, 25), Cliplimit (0.01). Other parameters were set to default. The image was then processed using the function of *imadjust* (stretchlim, output = 0 to 1, gamma = 0.6). The image was further processed by the linear filter and edge enhancement to enhance the vessel border (Fig. 3) [12]. We used the MATLAB function of *imfilter* [parameters: w = fspecial (average = 11, boundary options = replicate, others = default] to remove the artifact of the image edge, which was enhanced during the image contrast enhancement. This process will not affect the extraction of the vessels. In recent years, semantic segmentation methods have been used to segment the vessels from fundus photos, using deep learning approaches [13, 14]. One of the most representative networks is U-Net (convolutional neural network), which was developed for the biomedical imaging segment. This approach often uses the hand-labeled picture as the ground truth for the learning model. The U-NET deep learning method has been well described in previous studies [13–16]. In the present study, the U-NET structure based on the fully convolutional neural network [15] was developed and then used to enhance the contrast of vessels. After that, blood vessels were extracted (Fig. 3). The U-shaped network used 23 convolutional layers. The public datasets of digital retinal images for vessel extraction (Drive: https://www.isi.uu.nl/Research/Databases/DRIVE/) [13, 14, 17] were used as the training data. There were a total of 40 source images (565 × 584 pixels), which were used as training images. The random image patches were generated from the training image data and patched into the training image dataset [14]. A total of 38 blood vessel images were processed [13, 14]. The images were then converted to binary for vessel density analysis using ImageJ (Fig. 3). Vessel density was defined as the percentage of pixels within the vessels over the entire image.

### Statistical analysis

All values are expressed as mean ± standard deviation. Significance was assessed with the Student’s t-test for two variable comparisons using SPSS Statistics package (ver. 25, IBM Corp., Armonk, NY, USA). Pearson’s regression was used to determine the relationships among parameters. The Bland-Altman plot was constructed between the two measurements to determine the 95% limit of agreement, which was calculated as 1.96 × (the standard deviation of the difference between repeated measurements). *P* < 0.05 was considered statistically significant.

## Results

### Subject characteristics

Demographic information is shown in Table 1. Three participants were male, seven were female. Three study participants were healthy subjects, four had a history of diabetes mellitus (DM), and three had a history of multiple sclerosis (MS). In a total of 19 eyes, 10 eyes were right eyes, and 9 eyes were left eyes.

**Table 1:** Demographic information

	Mean ± SD	Range
**Age (years)**	49.5 ± 13.7	30–71
**SBP (mmHg)**	121.8 ± 19.5	101–162
**DBP (mmHg)**	77.5 ± 8.8	62–88
**HR (beats/min)**	75.2 ± 11.0	60–94

*SBP* = systolic blood pressures, *DBP* = diastolic blood pressures, *HR* = heart rate

### A/V ratio

**Fig. 4:**
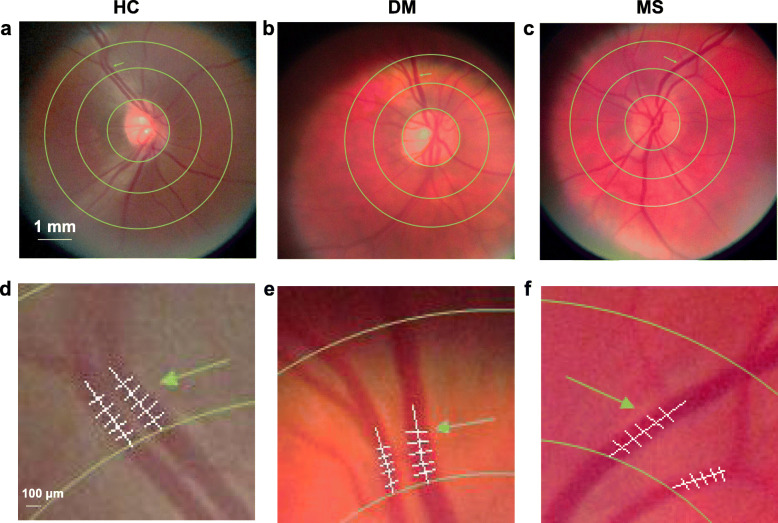
A/V ratio analysis of representative cases of healthy control (HC), diabetes mellitus (DM), and multiple sclerosis (MS) subjects. **a-c** Retinal photographs with circle defining the measurement. The arrow indicates selected parallel arteriole and venule. **d-f** Five non-overlapping diameter measurements of arteriole and venule by ImageJ

**Fig. 5:**
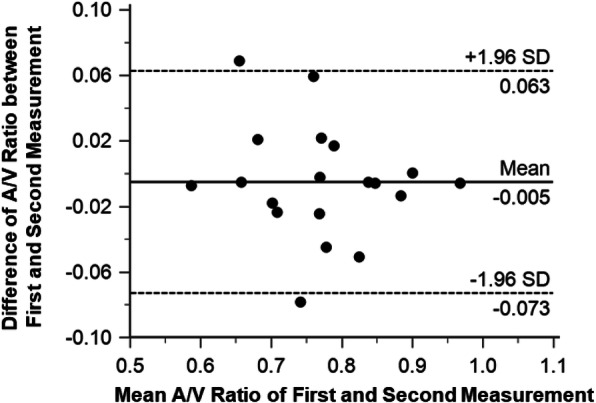
A/V ratio calculation of first and second measurements. Bland-Altman plot of differences in the first measurement vs. the second measurement

The A/V analysis was successfully processed in all subjects of each group (Fig. 4). There was no significant difference of averaged A/V ratio between the first (0.77 ± 0.09) and second (0.77 ± 0.10) measurements (*P* = 0.53) (Fig. 5). The A/V ratio of the first measurement ranged from 0.58 to 0.96, while it ranged from 0.59 to 0.97 in the second measurement. Two measurements were significantly correlated (*r* = 0.94, *P* < 0.001) (Fig. 5). Bland-Altman plot for the two measurements showed a bias of − 0.005 with upper and lower confidence intervals of 0.063 and − 0.073, respectively.

### Vessel density

**Fig. 6:**
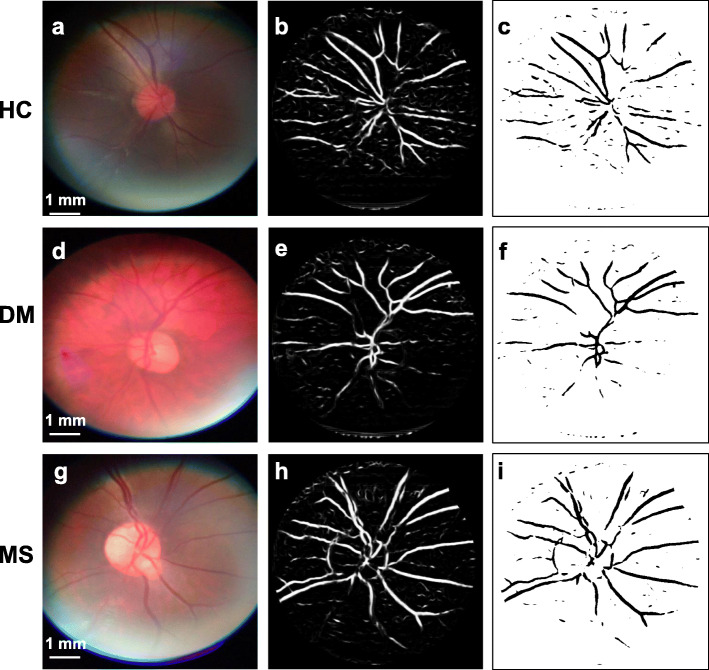
Vessel density analysis of representative cases of healthy control (HC), diabetes mellitus (DM), and multiple sclerosis (MS) subjects**. a**, **d**, **g** Adjusted square photographs. **b**, **e**, **h** Vessel extraction images. **c**, **f**, **i** Binary images

**Fig. 7:**
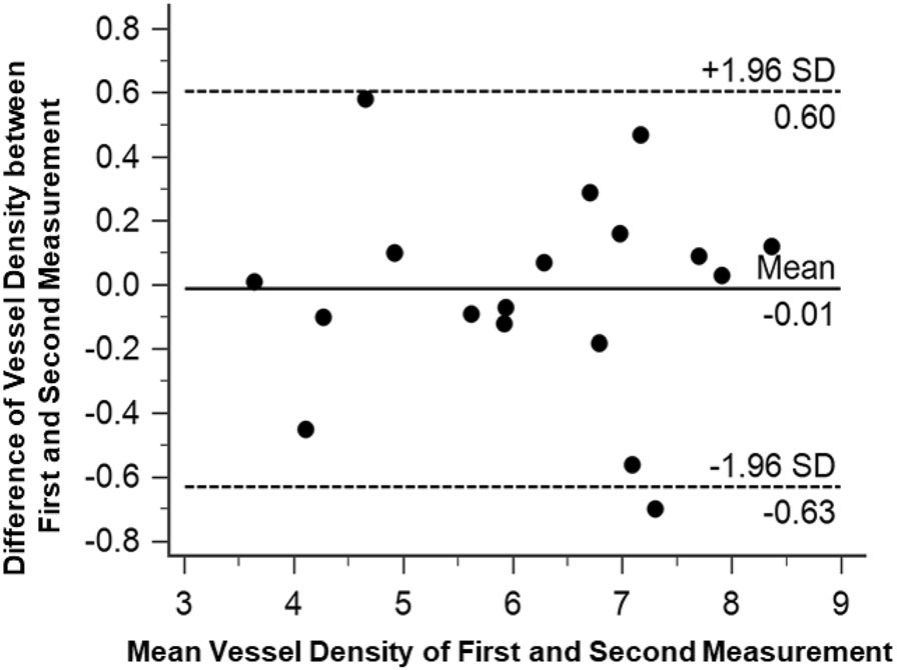
Vessel density analysis of first and second measurements. Bland-Altman plot of differences in the first measurement vs. the second measurement

Vessels were extracted from the fundus photos of subjects for analysis of vessel density (Fig. 6). There was no significant difference of averaged vessel density (%) between the first (6.11 ± 1.39) and second (6.12 ± 1.40) measurements (*P* = 0.85) (Fig. 7). In the first measurement, vessel density ranged from 3.64 to 8.42, while it ranged from 3.63 to 8.30 in the second measurement. The two measurements were significantly correlated (*r* = 0.97, *P* < 0.001). Bland-Altman plot for the two measurements showed no bias with upper and lower confidence intervals of 0.60 and − 0.63, respectively.

## Discussion

This study was to quantitatively analyze fundus photos for characterizing retinal vasculature by calculating the A/V ratio and vessel density in fundus photos taken using the PanOptic iExaminer system. This study provides evidence that fundus photos with sufficient quality for analysis of A/V ratio and vessel density, can be acquired using the portable PanOptic system for image analysis. The acquisition appeared to be facilitated by the modifications used in the present study. First, the ophthalmoscope was stabilized by the use of the slit-lamp base, which also made it easy to align with the eye. Second, the patient’s head was stabilized by resting on the chinrest. Third, the fixation target facilitated the gaze direction for imaging. Fourth, pupil dilation also helped in image acquisition. It may be worth noting that while these modifications may not alter the portability and data transfer, the slit-lamp base with the chinrest can be easily transported with the portable ophthalmoscope. The inconvenience of the use of the slit-lamp base may not outweigh the gain of easy acquisition of high-quality images for quantitative analysis. It is also worth noting that some of the inexpensive handheld fundus cameras are also available, although some of these cameras do not use smartphones [18, 19]. Based on the outcomes in the present study and previous studies [20], the PanOptic system could potentially be used for pre-screening in remote communities. It would be recommended that fundus photos may be analyzed using analysis software installed in the smartphone [20] or in fundus photo reading centers or research labs.

Previous studies (Table 2) demonstrated the usefulness of the PanOptic ophthalmoscope, while the present study provided an alternative solution to use the device with the add-on translator and chinrest. The PanOptic ophthalmoscope was used qualitatively for patients’ fundus imaging in the emergency room [23] and optic disc assessment [24], for diabetic retinopathy screening [7, 9] and medical students ophthalmoscopy skills assessment [8]. There are some studies using the PanOptic device, quantitatively analyzing the vessel diameter in the retina [20] and anterior lens capsule vascularity only [22]. In addition, vessel segmentation and analysis based on fundus photos taken with the PanOptic ophthalmoscope were also done in previous studies [20, 21]. Xu et al. did not analyze the A/V ratio and vessel density [20, 21], which are critical to the clinical applications of the PanOptic ophthalmoscope equipped with a smartphone. In addition, Xu et al. did not analyze the repeatability of these important measurements (i.e., A/V ratio and vessel density) [20, 21]. Our work focused on the characterization of retinal vasculature by quantitatively analyzing A/V and vessel density, which provides insightful information on the repeatability before testing whether the system can be used for clinical diagnosis. Although these previous studies [20, 21] and the present study applied a similar methodology to extract the vessel information, this study provides additional information to the feasibility and repeatability of measuring A/V ratio and vessel density. In addition, the alternative solution by adding the slit-lamp base may facilitate translating the portable ophthalmoscope equipped with the smartphone, such as the PanOptic device. However, more work needs to be done to test whether the portable ophthalmoscope equipped with the smartphone can differentiate diseased populations from the normal population.

**Table 2:** Summary of PanOptic ophthalmoscopy studies

Authors	Subjects	Subjects No.	Focus Area	Pupil Dilation	Smartphone	App	Mounted	Main outcome
Xu et al. 2016 [20]	Normal	10	Retina	Not mentioned	Android	iExaminer and Android app	No	Segment retinal vessels, analyze vessel width, and store or uplink results
Xu et al. 2018 [21]	Normal	10	Retina	Not mentioned	Not mentioned	Not mentioned	Not mentioned	Segment retinal arterioles and venules
Patel et al. 2019 [22]	Preterm infants	24	Anterior lens capsule vascularity	No	iPhone 4 & 6 s	MoviePro	Ring stand	Quantitative analysis for gestational age estimate
Day, et al. 2017 [23]	Pediatric emergency patients	184	Retina	No	Not mentioned	iExaminer	Not mentioned	Feasibility of fundus photography in pediatric patients
Petrushkin et al. 2012 [24]	Emergency patients	36	Optic disc	No	No photography	No	No	More sensitive and specific than the direct ophthalmoscope
Tan et al. 2010 [9]	Diabetes mellitus	200	Retina	Yes	No photography	No	No	Not superior to direct ophthalmoscope for retinopathy
Gill et al. 2004 [7]	Diabetes mellitus	28	Retina	No	No photography	No	No	Fairly accurate in screening diabetic retinopathy
McComiskie et al. 2004 [8]	Healthy volunteers	140	Optic disc	No 75, Yes 65	No photography	No	No	Easier to use, with the accuracy of rating the cup to disc ratio
Desai et al. 2018 [25]	Neonates	124	Anterior lens capsule vasculature	No	iPhone 6 Plus	iExaminer	No	Gestational age estimation
Lee et al. 2020 [26]	Healthy volunteers	Not mentioned	Optic nerve head	No	No photography	No	No	Ophthalmology clinical training
Besenczi et al. 2015 [27]	Normal	16	Retina	Not mentioned	iPhone 4/4S	iExaminer	No	Automatic optic disc and optic cup detection

Compared to images captured by traditional fundus cameras and Ultra-wide field scanning laser ophthalmoscopy (SLO), the resolution (1280 × 720 pixels in video recording) of PanOptic fundus images is not high due to the settings in the iExaminer software for video recording. However, there are some advantages to making it a practical instrument for retina blood vessel analysis of disease screening (Table 3), which are its portability, low cost, smartphone adaptability, and easy data transferring. Traditional fundus camera offers good-quality images but is bulky, office-based, and technician dependent, which limits its use as a community screening tool, especially in remote areas. SLO, with a 180–200° field of view, offers faster and easier image acquisition without pupil dilation when compared to traditional fundus cameras [28]. It is ideal for a hospital-based study. However, SLO is costly, which may reduce its availability for disease screening. In our future study, we will compare the retinal vascular network analysis results of the PanOptic iExaminer system with those of traditional fundus cameras and SLO.

**Table 3:** Comparison of fundus imaging systems

	Direct Ophthalmoscope	PanOptic iExaminer System	Traditional Fundus Camera (Topcon TRC-NW8F)	Ultrawide field SLO (Optos California af)
Field of View	5°	25° - 30°	45°	200°
Resolution	N/A	720 × 1280 pixel	16.2 megapixel	14 μm
Portability	Yes	Yes	No	No
Cost	Low	Low	High	Very High
Smart Phone Adapted	No	Yes	No	No
Availability for Screening	Yes	Yes	No	No

The A/V ratio acquired using the PanOptic iExaminer system appeared within the range of those found at using the traditional fundus camera of previous large sample studies [10, 29, 30]. The good repeatability of this analysis indicated its feasibility using the analysis method of McClelland et al. [31] although in this study, the A/V ratio’s correlation to other disease conditions was not analyzed as the focus of this paper was a feasibility test. Other studies already reported that the A/V ratio of the retina is a widely used parameter in the assessment of different ocular and systemic vascular diseases, such as open-angle glaucoma [32], incident stroke [32], coronary heart disease [33] and dementia [34].

There were several limitations to this study. First, the aim of this study was to characterize retinal vasculature using the commercially available ophthalmoscope equipped with a smartphone by reporting the feasibility and repeatability of the measurements. Although this study provided an alternative solution for resource-limited regions and countries, there are low-cost portable handheld fundus cameras such as the portable handheld camera with a retinal model (Pictor, Volk Optical, Inc., Mentor, OH, USA) [18, 35]. The camera could perform a similar task for pre-screening [18, 35]. Therefore, more work will need to be done to test whether the portable ophthalmoscope equipped with the smartphone, such as the PanOptic ophthalmoscopy, can be used to screen for retinal diseases. Second, although we aimed for the feasibility of analysis, the sample size was still small. We could not compare the differences among groups. Larger sample size and case-control studies are needed to further verify further whether the described method could differentiate vasculature alterations in the pathologic retina. Third, adding the slit-lamp translator and chin-and-head rest as an alternative add-on of the PanOptic ophthalmoscope will increase the cost, which amounts to an entry-level retinal camera. Nevertheless, our study provided an alternative solution to use the PanOptic ophthalmoscope with the translator and chinrest. Fourth, we did not compare the benefit of the smartphone to other portable fundus cameras without smartphones. While different approaches using the smartphone on the handheld fundus camera or ophthalmoscopes are available and continuously being developed, adding the smartphone may not necessarily make a device better. The applications using the smartphone on the portable fundus camera may be dependent on the availability and the need for particular functionalities.

## Conclusions

This study demonstrated the feasibility of characterizing retinal vasculature using the PanOptic iExaminer System, which yielded sufficient image quality of the fundus photos for quantitative analysis of A/V ratio and vessel density. The device appears to provide the benefit of portability, easy data transferring, and cost-effectiveness for the possible use in pre-clinical screening of systemic, cerebral, and ocular diseases.

## Data Availability

The datasets used and analyzed for the present study are available from the corresponding author upon reasonable request.
